# Analyzing the G3BP-like gene family of *Arabidopsis thaliana* in early turnip mosaic virus infection

**DOI:** 10.1038/s41598-021-81276-7

**Published:** 2021-01-26

**Authors:** Hendrik Reuper, Khalid Amari, Björn Krenz

**Affiliations:** 1 Leibniz Institute DSMZ-German Collection of Microorganisms and Cell Cultures, Inhoffenstr. 7 B, 38124 Braunschweig, Germany; 2 Julius Kühn Institute (JKI) - Federal Research Centre for Cultivated Plants, Institute for Biosafety in Plant Biotechnology, Erwin-Baur-Str. 27, 06484 Quedlinburg, Germany

**Keywords:** Plant sciences, Plant stress responses, Abiotic, Biotic

## Abstract

The *Arabidopsis thaliana* genome encodes several genes that are known or predicted to participate in the formation of stress granules (SG). One family of genes encodes for Ras GTPase-activating protein–binding protein (G3BP)-like proteins. Seven genes were identified, of which one of the members was already shown to interact with plant virus proteins in a previous study. A phylogenetic and tissue-specific expression analysis, including laser-dissected phloem, by qRT-PCRs was performed and the sub-cellular localization of individual AtG3BP::EYFP fluorescent fusion proteins expressed in *Nicotiana benthamiana* epidermal cells was observed*.* Individual AtG3BP-protein interactions *in planta* were studied using the bimolecular fluorescence complementation approach in combination with confocal imaging in living cells. In addition, the early and late induction of G3BP-like expression upon Turnip mosaic virus infection was investigated by RNAseq and qRT-PCR. The results showed a high divergence of transcription frequency in the different plant tissues, promiscuous protein–protein interaction within the G3BP-like gene family, and a general induction by a viral infection with TuMV in *A. thaliana*. The information gained from these studies leads to a better understanding of stress granules, in particular their molecular mode of action in the plant and their role in plant virus infection.

## Introduction

The Ras GTPase-activating protein-binding protein (G3BP), well investigated in humans and animals, is a member of the heterogeneous nuclear RNA-binding proteins and an element of the Ras signal transduction pathway. It was originally reported to bind to the Ras-GTPase-activating protein by associating with its SH3 domain, which was name-giving, but recent findings showed a central role of G3BP in translational control and RNA stability^1^. In humans, the G3BP protein family is comprised of G3BP-1, G3BP-2a, and its splice variant G3BP-2b. All G3BPs share four distinct motifs: (1) a nuclear transport factor 2 (NTF2)-like domain at the N-terminus, (2) an acidic and proline-rich region in the central part of the protein, (3) an RNA recognition motif (RRM) followed by (4) an arginine and glycine-rich region (RGG domains) at the C-terminus. The NTF2-like domain is involved in nuclear transport of G3BP-1 and, most likely, also G3BP-2^1^. Moreover, the NTF2-like domain has been shown to facilitate protein–protein interactions; thus, G3BP interacts with several proteins but also forms homodimers^2^ or oligomers^3^. G3BP interacts with several proteins^4–6^, most interestingly with CAPRIN1 via its NTF2-like domain^7^ or with USP10^8^. For CAPRIN1 no plant homolog protein has been identified yet, but the ubiquitin-specific protease 24 from *Arabidopsis thaliana* (AT4G30890) has striking sequence similarity with HsUSP10. Indeed, a AtG3BP-like protein interacts with AtUBP24 in bimolecular fluorescence complementation (BiFC) experiments^9^. Taken together, G3BP is a multifunctional protein, which interacts with several other cellular proteins at different developmental stages in different cell types, either in human or animal cells. However, the precise biological function(s) of G3BP has not been elucidated yet, but it is suggested that G3BP is involved in neurological diseases^10,11^. More recently, the overexpression of G3BP-1 has been implicated in cancer, suggesting a functional role of G3BP-1 in tumorigenesis^12–14^. In addition, G3BP is an important component of the host antiviral defense^15,16^. The general opinion is that G3BP executes its role by stress granule (SG) formation to regulate cellular homeostasis, RNA metabolism, and gene expression at the posttranscriptional level^17,18^. For example, human G3BP-1 can initiate SG formation^2^, which causes clustering of mRNA molecules derived from polysomes into SGs via a mechanism that involves G3BP and other proteins to halt translation. This makes SGs a primary target of virus infection. Viruses can employ different strategies to interfere with SG assembly^16,19–21^. For example, the non-structural protein 3 (nsP3) of Semliki Forest virus (SFV) can bind to the NTF2-like domain of G3BP-1 via an ‘FGDF’ motif and thereby preventing SG assembly and promoting viral RNA translation^22–24^ or multiple poliovirus proteins affect RNA granules to varying extents, G3BP-1, for example, is cleaved by 3C^pro^^25^.

The general pathway of SG formation and function seems to be conserved, but only a few studies about plant stress granules are available^18,19^ compared to mammalian stress granules. For example, the G3BP-like family in *A. thaliana* has not yet been fully characterized. Abulfaraj and colleagues^26^ describe eight family members, generated and analyzed AtG3BP-7 (AT5G48650) OEX lines and KO lines, which showed no phenotype compared to control plants. This might be due to the fact that G3BPs in *A. thaliana* are redundant in their function, and a KO of one AtG3BP could be compensated by one of the others. Nevertheless, G3BP in plants and its interactions with plant viruses are poorly understood, although 'FGDF'-like binding motifs can be found in some plant viruses^22^, for example, it has been shown that the helper component proteinase (HC-pro) of potato virus A (PVA) induces the formation of RNA granules^27^. Only a few studies are investigating the interaction between plant viruses and the host's G3BP homologue, only recently, it was shown that *A. thaliana* G3BP-2 interacts with the nuclear shuttle protein (NSP) of the plant virus Abutilon mosaic virus^9^, but the purpose of this interaction is still unknown.

Turnip mosaic virus (TuMV), a member of the family *Potyviridae*, belonging to genus *Potyvirus*, has a single-stranded RNA genome. In general the size of the genomic RNA is < 10 kb and encodes a polyprotein, including the P1 protease^28^. At the N-terminal end of the P1 of Turnip mosaic virus (P1-TuMV) two FGDF-like motifs are located. This motif has been shown to bind the host protein G3BP^22^. This suggests that also P1-TuMV interacts with G3BP in plants in a similar matter. Aim of this study was therefore to gain more information about all putative members of G3BP-like proteins in *A. thaliana*, i.e. in terms of tissue expression, cellular localization and protein–protein interaction, and to investigate the response of the AtG3BPs to a viral infection, i.e. TuMV. Special emphasis here was also to monitor AtG3BPs expression to an early time point of infection.

## Results

### Phylogenetic analysis

**Figure 1:**
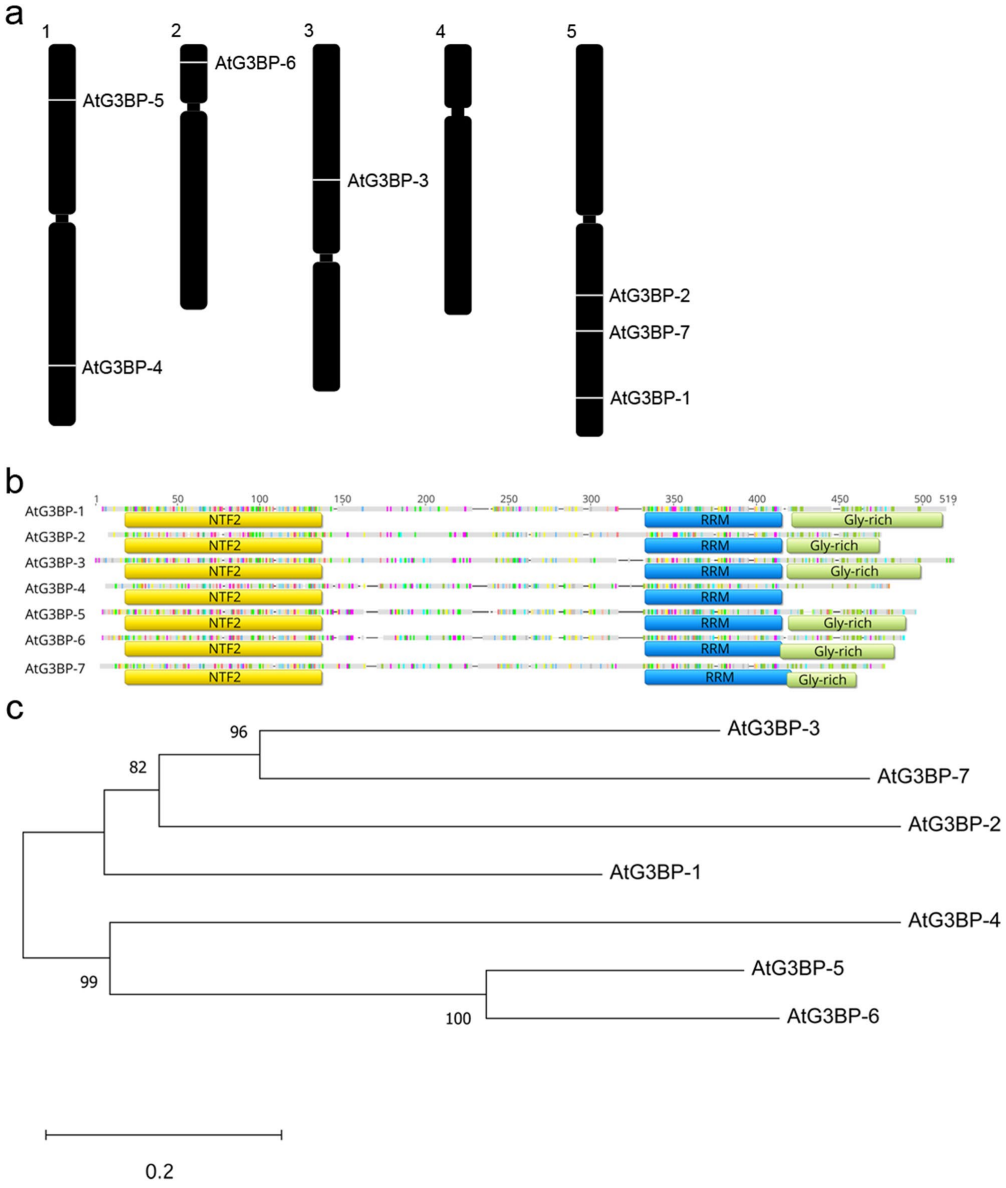
Relationship of the G3BP-like gene family in *A. thaliana*. (**a**) Genomic location of the different G3BP-like gene family in *A. thaliana*. The image was drawn using Adobe Photoshop CS4 (https://www.adobe.com/de/products/photoshop.html). (**b**) Schematic alignment of the seven G3BPs from *A. thaliana* generated with Geneious Prime® 2020 software. Nuclear transport factor 2 (NTF2), RNA-binding (RRM), and Gly-rich domains are annotated and highlighted with yellow, blue and green boxes, respectively. (**c**) The evolutionary history was inferred as described in the methods section. The optimal tree with the sum of branch length = 3.69183953 is shown. The tree is drawn to scale, with branch lengths in the same units as those of the evolutionary distances used to infer the phylogenetic tree. This analysis involved 7 amino acid sequences. All ambiguous positions were removed for each sequence pair (pairwise deletion option). There were a total of 524 positions in the final dataset.

Bioinformatic analysis of the *A. thaliana* genome (TAIR10) revealed seven possible members of an *A. thaliana* G3BP-like gene family. These candidates are distributed on different chromosomes of the *A. thaliana* genome (Fig. 1a). AtG3BP-1, -2, and -7 are located on chromosome 5, AtG3BP-3 on chromosome 3, AtG3BP-4, and -5 on chromosome 1 and AtG3BP-6 on chromosome 2. They all have been identified by and thus share an N-terminal NTF2-like domain and a C-terminal RRM domain. Furthermore, all of them harbor the Gly-rich region and RG(G) motifs at the C-terminus, except AtG3BP-4 (Fig. 1b). Additionally, they contain several conserved amino acids whose functional relevance has been demonstrated in the human homolog, such as phenylalanine at position 41 of the consensus sequence (equivalent to position 33 in HsG3BP-1), which all AtG3BPs, again with the exception of AtG3BP-4, share with their mammalian homolog. A protein sequence alignment was performed to show the intra-family relatedness between the postulated members (Fig. 1c). They cluster into two main groups, the first one consisting of AtG3BP-1, -2, -3, and -7, the second one consisting of AtG3BP-4, -5, and -6.

Sequence comparison of each of the three different AtG3BP domains showed no significant alteration to the phylogenetic analysis based on the full amino acid sequences comparison. AtG3BP-1, -2, -3, and -7, and AtG3BP-4, -5, and -6 still cluster together if only the NTF2 domain is analyzed (Supplementary Fig. S1a) and with minor changes if the RRM domain is compared (Supplementary Fig. S1b). Strikingly, AtG3BP-1 and -7, and -2 and -3 cluster together, when comparing only the Gly-rich regions (Supplementary Fig. S1c), because AtG3BP-4 lacks this region. The cluster AtG3BP-5 and -6 remains unchanged. Table 1 summarizes the protein sequence similarity and the nucleotide sequence identity of the coding sequences based on the ClustalW sequence alignment. Here, AtG3BP-5 and AtG3BP-6 show the highest amino acid sequence identity, 67%, whereas AtG3BP-2 and -5 the lowest with 36.2%.

**Table 1:**
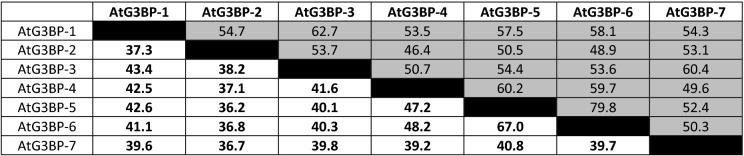
Sequence similarity of the different AtG3BPs in %.

The protein sequence similarity (shaded in grey) and the nucleotide sequence identity (white boxes) of the coding sequences were calculated based on the ClustalW alignment with the BLOSUM62 matrix and a threshold of 0.

### Subcellular localization of AtG3BP::EYFP fusion proteins in *N. benthamiana*

**Figure 2:**
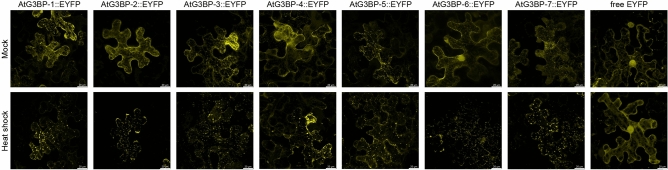
Subcellular localization of AtG3BP proteins and free EYFP in transient expressing *N. benthamiana* leaves under ambient conditions and after heat shock. Pictures are maximum projections of z-stacks obtained by confocal laser scanning microscopy and were taken 2 dpai. The individual pictures correspond to a size of 145 × 145 µm.

To study the subcellular localization, the AtG3BPs were each transiently overexpressed in functional fusion with enhanced yellow fluorescent protein (EYFP) under the control of the CaMV 35S promoter in *N. benthamiana* epidermis cells (Fig. 2). All possible members of the AtG3BP family showed a cytoplasmic signal under ambient condition, with AtG3BP-6 also a nuclear EYFP signal in addition. Furthermore, AtG3BP-3, -5, and -7 form granule-like structures already under ambient condition. After heat shock treatment, these structures can be observed also for the other AtG3BPs (Fig. 2). The nuclear EYFP signal of AtG3BP-6 can still be observed after stress application (Supplementary Fig. S2). The expression of the fusion proteins was verified by western blot analysis (Supplementary Fig. S3).

### AtG3BP protein interaction profiling in vivo

**Figure 3:**
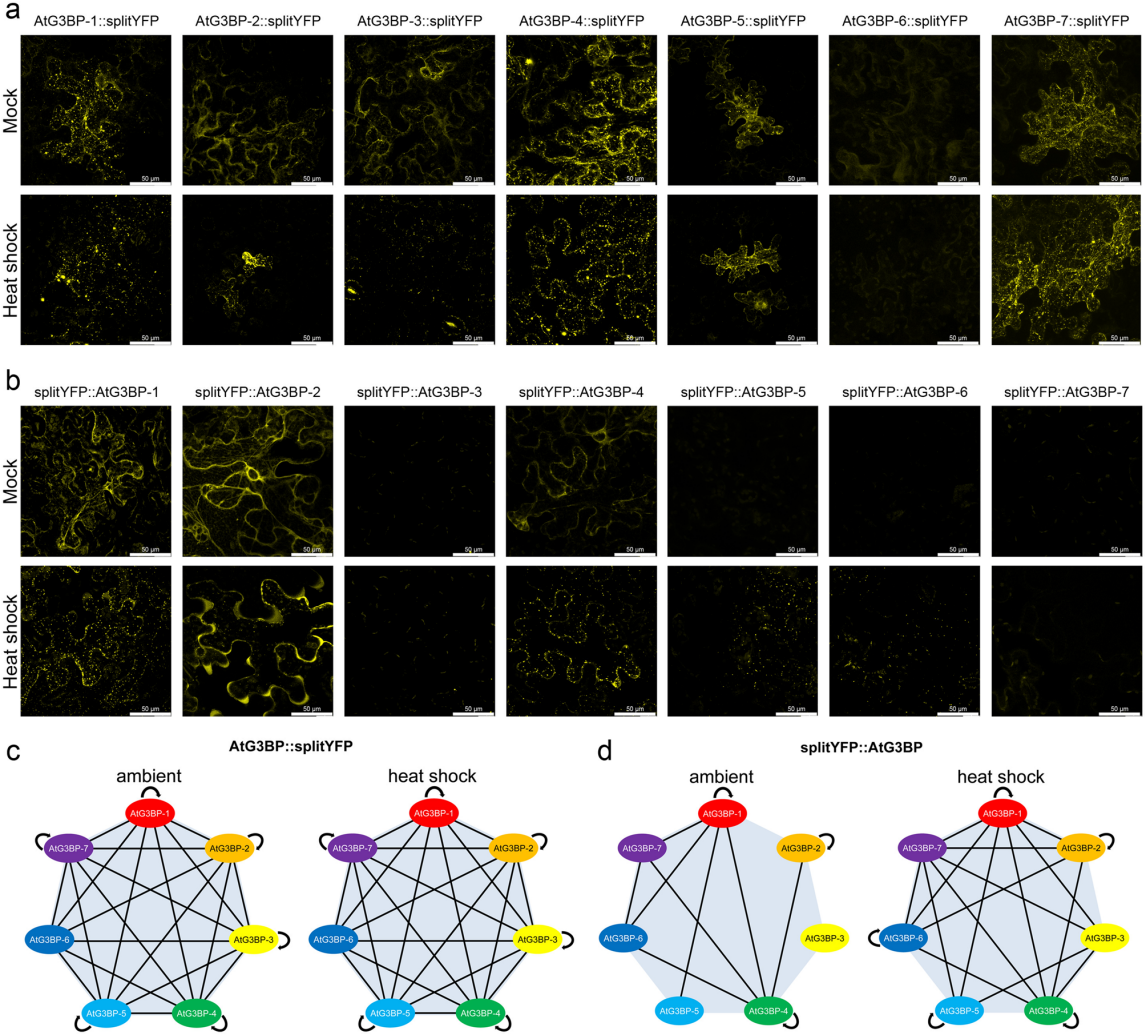
Visualization of self-interaction of the various AtG3BPs via bimolecular fluorescence complementation (BiFC) under ambient conditions and heat shock (45 min at 37 °C). (**a**) Self-interaction of *A. thaliana* G3BP homologs fused C-terminally with splitYFP. (**b**) Self-interaction of the respective AtG3BPs N-terminally fused to splitYFP. All pictures are projections of z-stacks obtained by confocal laser scanning microscopy. Fusion constructs of the protein with the corresponding reporter were transiently expressed in *N. benthamiana* leaves by co-infiltration via *A. tumefaciens* at an OD_600_ = 0.1. The individual pictures correspond to a size of 185 × 185 µm. (**c**,**d**) Schematic overview of the protein–protein interactions between the different AtG3BPs. Proteins were fused C-terminally (**c**) or N-terminally (**d**) to splitYFP and pairwise co-expressed in *N. benthamiana* leaves. Cells were checked for re-constituted YFP signal 2 dpai and heat shock was performed to examine changes of interaction under stress conditions. Schematic overviews were created using Microsoft PowerPoint 2010 software.

Previous studies have shown that the NTF2-domain plays a key role in the oligomerization of HsG3BP-1^2^. Since all predicted *A. thaliana* G3BP homologs share this domain, we analyzed their ability to form homo- and heterooligomers by BiFC. All 49 pair-wise combinations were co-expressed in *N. benthamiana* leaves and monitored for a reconstituted fluorescent YFP signal under ambient conditions and after heat shock treatment. The data for homooligomerization is shown in Fig. 3a and b, and for heterooligomerization summarized in Fig. 3c and d, pictures in the Figs. S4–S7. Figure 3a shows the reconstituted YFP signals for the different AtG3BPs C-terminally fused to the respective YFP fragment before and after heat shock. All AtG3BPs but AtG3BP-6 showed cytoplasmatic YFP signal under ambient conditions, with AtG3BP-1, -4, -5, and -7 already forming granule-like structures under these conditions. After heat shock, the evenly distributed cytoplasmic-localized YFP fluorescence changed almost completely into a granular YFP signals. AtG3BP-6 C-terminally fused to splitYFP did not show any signal after heat shock. When fused N-terminally to the respective YFP fragments, only AtG3BP-1, -2, and -4 showed an exclusively cytoplasmatic reconstituted YFP signal. After heat shock treatment YFP fluorescent, granule-like structures can be observed for AtG3BP-1, -2, -4, -5, and now also for -6. We also tested the ability of the different AtG3BPs to form heterodimers in vivo with each other by co-infiltrating the reciprocal splitYFP fusion proteins and examining for reconstituted YFP signals two days post agroinfiltration (dpai) by laser scanning microscopy (Figs. S4–S7). The results are summarized in Fig. 3c and d. Overall, all AtG3BPs seem to interact with each other under ambient conditions as well as stress conditions (in this case heat shock).

**Figure 4:**
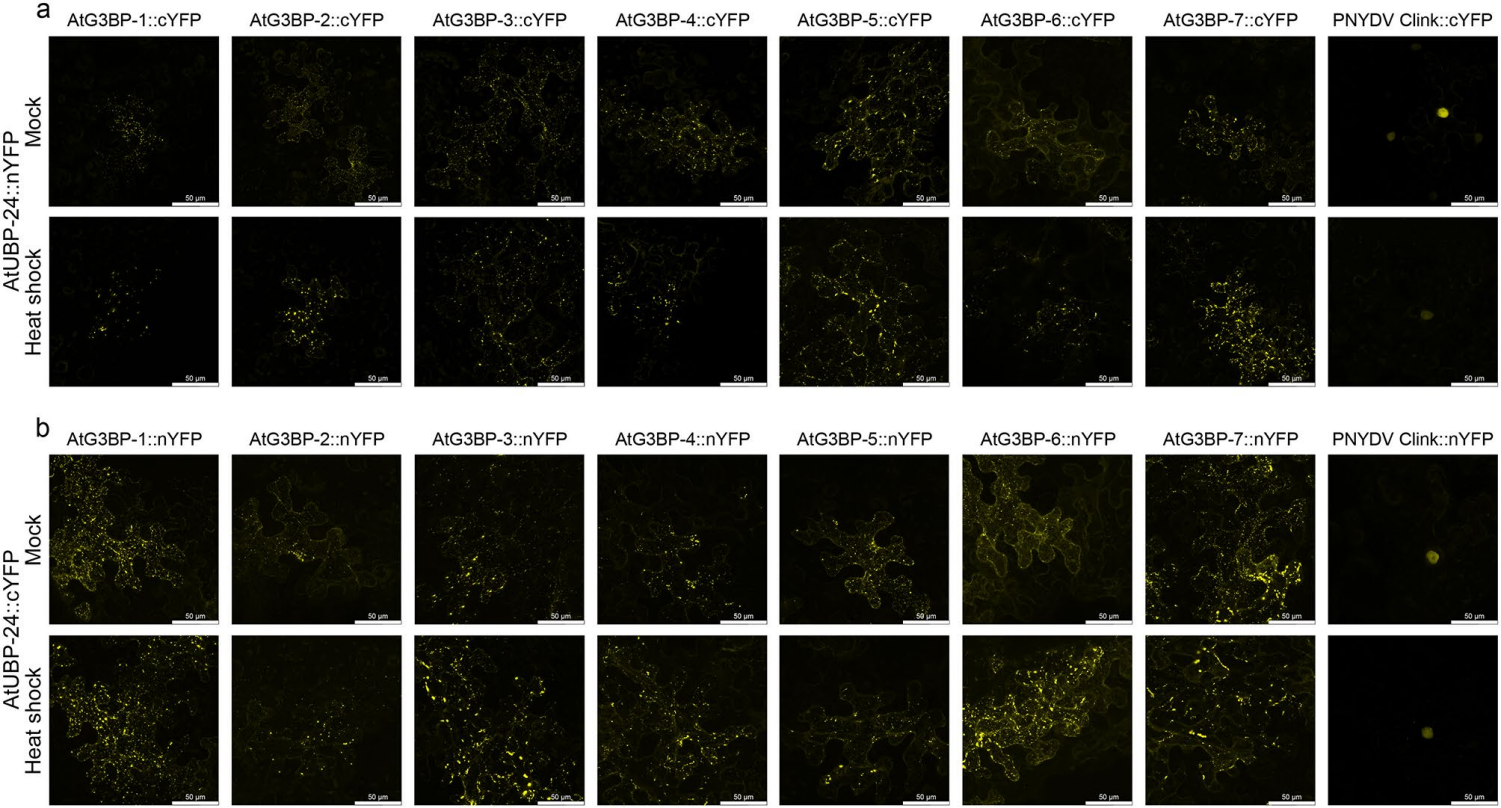
Visualization of protein–protein interaction of the AtUBP-24 with the various AtG3BPs and PNYDV Clink via bimolecular fluorescence complementation (BiFC) under ambient conditions and heat shock (45 min at 37 °C). (**a**) Interaction of AtUBP-24 with the corresponding partners fused C-terminally with splitYFP. (**b**) Interaction of AtUBP-24 and the respective AtG3BPs and PNYDV Clink N-terminally fused to splitYFP. All pictures are projections of z-stacks obtained by confocal laser scanning microscopy. The individual pictures correspond to a size of 185 × 185 µm.

**Figure 5:**
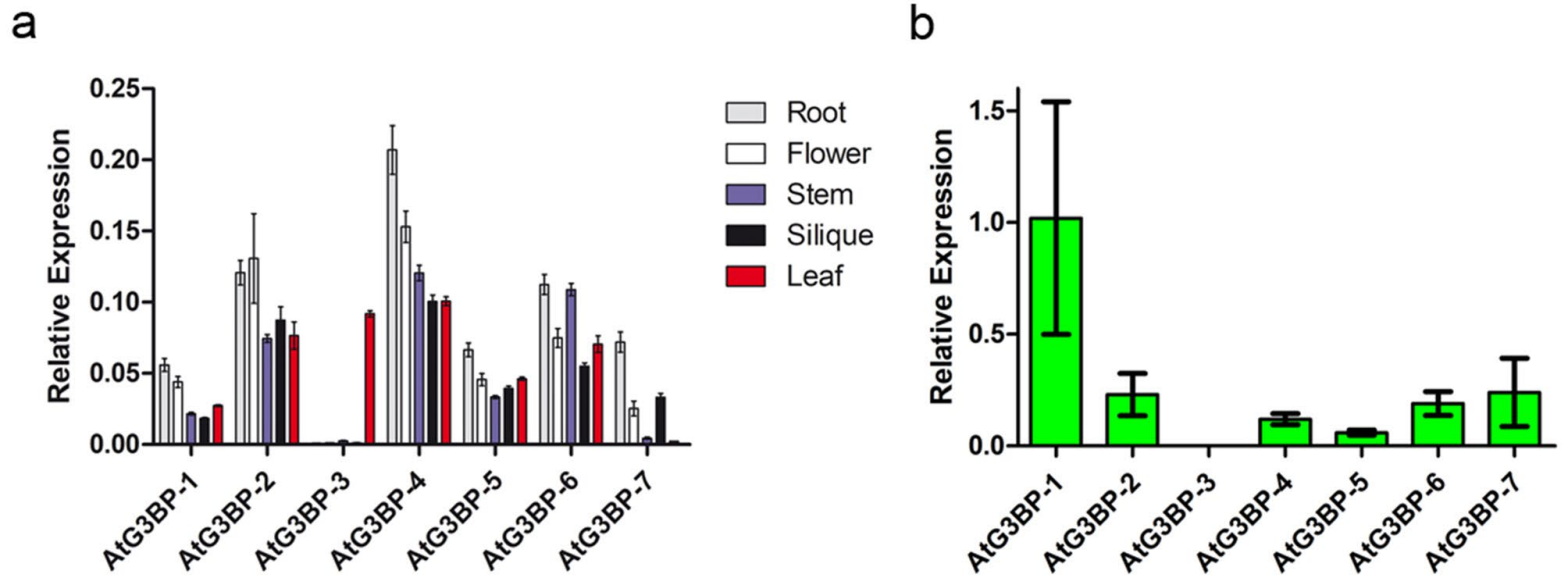
Expression level analyses. (**a**) qRT-PCR was used to measure the relative gene expression of the different *AtG3BP*s in roots, stem, flower, silique, and rosette leaves. Transcript levels were calculated using the 2^-∆Ct^ method with *AtActin-2* as the endogenous control. The error bar represents the standard error. (**b**) Expression ratio of *AtG3BP* genes in phloem tissue compared to non-phloem tissue in *A. thaliana* leaves extracted by laser dissection of *AtSUC2*promoter::GFP plants. Gene expression was normalized to *AtActin-2* and expression levels were calculated using the 2^−∆Ct^ method.

To test whether the observed granule-like structures are indeed stress granules, we performed BiFC experiments with the stress granule protein AtUBP-24 (AT4G30890)^9^. As a control, we used the Clink protein of the Pea necrotic yellow dwarf virus (Accession JN133280) to show that our predicted AtG3BPs actually promote stress granule formation and not AtUBP-24 alone (Fig. 5). AtUBP-24 harbors an ‘FGSF’-motif at its N-terminal end, similar to the ‘FGDF’-motif of HsUSP-10, the protein’s G3BP binding domain. C-terminal fusion constructs of AtG3BP or Clink with cYFP were co-agroinfiltrated into *N. benthamiana* leaves with AtUBP-24::nYFP (Fig. 4a), AtG3BP::nYFP and Clink::nYFP with AtUBP-24::cYFP (Fig. 4b), respectively. Epidermal cells were monitored for reconstituted YFP signals at two dpai by confocal microscopy. All combinations of the different AtG3BPs with AtUBP-24 showed granular YFP signal at ambient conditions and after heat shock, whereas co-expression of Clink with AtUBP-24 showed an exclusively nuclear signal for both conditions.

### qRT-PCR analysis of AtG3BP gene transcript abundance

The presence of seven *A. thaliana* G3BP-like homologs raises the question of their distinct function(s), the possibility of redundancy in function(s) in either the same or in different tissues and their possible role in virus infection^9^. To investigate the expression levels a qRT-PCR analysis of transcript abundance has been performed for G3BP-like gene family members in different plant tissue, namely root, stem, flower, silique, leaf, and phloem under ambient conditions. Except for *AtG3BP-3* and *AtG3BP-7*, the different G3BP genes show a ubiquitous expression throughout all tested tissues. While *AtG3BP-3* shows expression only in leaf tissue, AtG3BP-7 is nearly not detectable in leaves, but primarily in root tissue. Overall expression levels between the different AtG3BPs were comparable, with *AtG3BP-4* showing the highest constitutive expression level (Fig. 5a).

Phloem tissue from a transgenic GFP driven under the *AtSUC2* promoter *A. thaliana* plant was sampled utilizing a laser dissection microscope (Supplementary Video S1) to monitor AtG3BPs transcripts abundance in this specialized tissue. With the exception of *AtG3BP-3*, all AtG3BPs were detectable in the phloem. *AtG3BP-1* shows the highest relative expression, followed by *AtG3BP-2, -6,* and -*7*. *AtG3BP-4* and *-5* are the lowest expressed in the phloem compared to the other AtG3BPs (Fig. 5b). A direct comparison between the relative expression values in the phloem tissue and the other tissues might be incorrect, due to the differences in the RNA extraction procedure. Petioles were cut, fixed, sectioned, and laser-dissected to gain phloem tissue. Nevertheless, an expression level analyses of the different *AtG3BP*s transcripts in roots, stem, flower, silique, and rosette leaves, which includes the data from phloem tissue, is shown in Supplementary Fig. S8.

**Figure 6:**
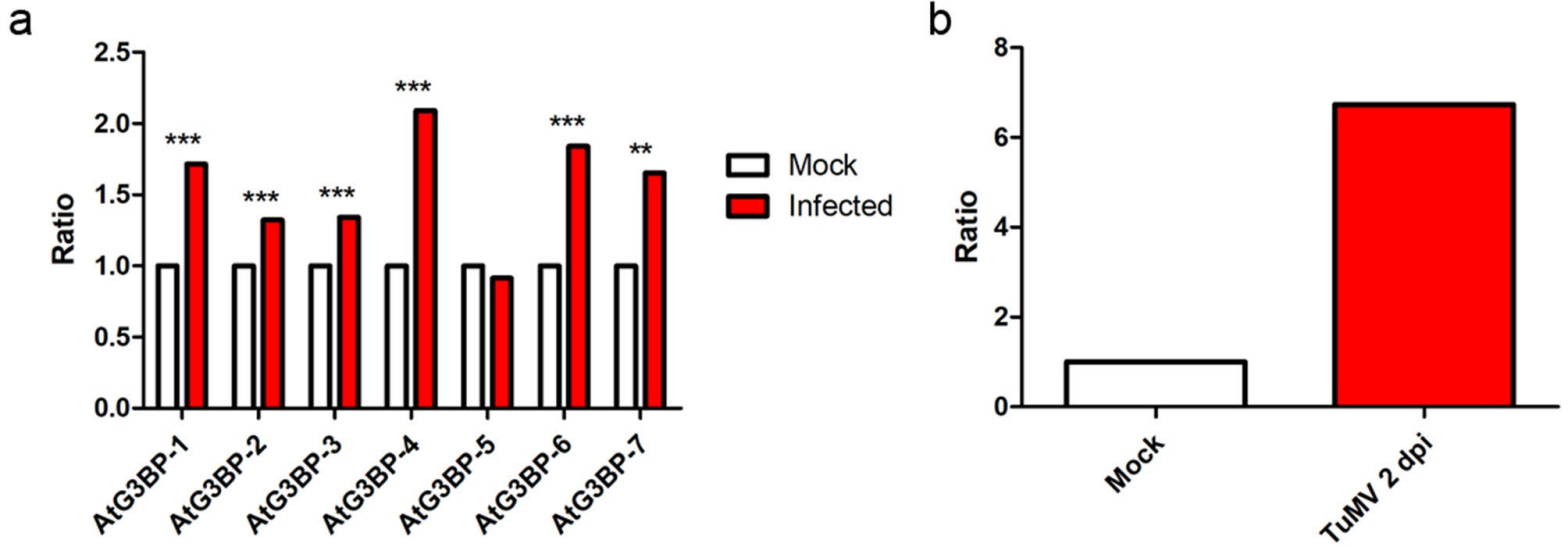
Expression level analyses. (**a**) Transcript abundance in TuMV-infected leaf tissue was measured and compared to non-infected tissue to determine the regulation of the expression level of the different AtG3BPs, *AtActin-2* served as the internal control. The asterisks indicate significance measured by two-tailed t-test (** = *p* < 0.01; *** = *p* < 0.005) (**b**) qRT-PCR analysis for verification of RNAseq data. *AtG3BP-4* expression level in TuMV infection sites 2 dpi was measured and compared to mock-inoculated tissue.

AtG3BP-2 was shown to interact with the nuclear shuttle protein of a plant virus^9^, which raised the question if the expression levels of the different *A. thaliana* G3BP-like homologs are affected by a plant virus in *A. thaliana*, for example TuMV. TuMV, which also expressed a RFP fused with a nuclear localization signal (TuMV-RFP^29^) was used in further studies. Microscopic analysis confirmed systemic infection of *A. thaliana* plants and RFP expression (Supplementary Fig. S9). TuMV-RFP infection generally induced AtG3BPs expression in the leaf (Fig. 6a) at 14 dpi, significantly, except AtG3BP-5.

The success or failure to establish an infection of a plant by a pathogen depends on an early cascade of molecular interactions between the plant and the pathogen in the initially infected cells. To study the early events of virus infection in plants, avoid dilution effects and thus to obtain the most meaningful information, micro-dissected tissue at 2 dpi was collected and subsequently analyzed in a transcriptomic approach (RNAseq) (Table 2) and subsequently by qRT-PCR (Fig. 6b).

**Table 2:** RNAseq data for the different AtG3BPs and AtUBP-24 in TuMV-RFP infection sites at 2 dpi compared to the mock-treated tissue.

Gene ID	Gene name	log2 FC	*p* value	P-adj
AT5G60980	*AtG3BP-1*	0.483	0.496	NA
AT5G43960	*AtG3BP-2*	1.022	0.269	NA
AT3G25150	*AtG3BP-3*	− 0.022	0.983	NA
AT1G69250	*AtG3BP-4*	1.711	0.009	0.060
AT1G13730	*AtG3BP-5*	− 0.174	0.867	NA
AT2G03640	*AtG3BP-6*	0.063	0.951	NA
AT5G48650	*AtG3BP-7*	− 0.387	0.694	NA
AT4G30890	*AtUBP-24*	0.175	0.849	NA

The different AtG3BPs were unevenly expressed, either up- or down-regulated, with the striking feature that AtG3BP-4 most probably significantly upregulated (1.7fold), which was then confirmed by qRT-PCR analysis (Fig. 6b). AtG3BP-4 was induced sixfold by TuMV infection at 2 dpi.

## Discussion

Human G3BP, first reported by Parker and colleagues^30^, binds to the SH3 domain of RasGAP. Responsible for the binding was the N-terminal nuclear transfer factor 2 (NTF2)-like domain^31^. The C-terminal portion of HsG3BP-1 contains an RRM motif, indicating that HsG3BP-1 has RNA-binding capacities. Indeed, HsG3BP-1 was reported to co-immunoprecipitated with mRNAs and to bind to and cleave, for example, the 3′ untranslated region of the c-myc mRNA in vitro^9^. Database searching has allowed the identification of members of the *A. thaliana* G3BP-like gene family. Seven G3BP-like proteins, within the group of NTF2 and RRM domain family proteins, in *A. thaliana* were identified and further investigated in this study. Abulfaraj and colleagues^26^ described additionally an RNA-binding (RRM-RBD-RNP motif) domain nuclear transport factor 2 family protein (AT3G07250), but in contrast to the other seven, AT3G07250 has a different domain structure, i.e., three RRM domains instead of one and the Gly-rich region and the RG(G) motifs are missing. In addition, the *A. thaliana* Information Resource (TAIR) database^32^ and Klepikova eFP RNAseq data revealed that AT3G07250 is only expressed in flowers 12–14^33^. Attempts to amplify the ORF of AT3G07250 by RT-PCR from leaf tissue consequently failed (data not shown), whereas transcriptional analysis showed that AtG3BP-1 to -7 were expressed, mostly throughout the plant, with the exception of AtG3BP-3. AtG3BP-3 amplification was negative in phloem tissue but exclusively expressed in leaf tissue, which might be a hint for guard cell-specific expression. Guard cells form stomata and are highly specialized cells, in which gene expression is often regulated by specific promoter activity^34^. Promoter studies will provide insight into plant cell-specific gene expression of the AtG3BP gene family in the future. In summary, it is a reasonable assumption that the different G3BP-like proteins within the G3BP gene family in *A. thaliana* have redundant function in different tissues at different developmental stages. It is therefore suggested that the AtG3BPs, like their animal counterparts, are likely to be involved in a wide variety of cellular processes, most importantly in stress granules formation. This suggestion is strongly supported by the fact that the two G3BP family members in mammals,, G3BP-1 and G3BP-2, and both proteins co-localize in SGs, when cells are subjected to stress^35,36^. Sub-cellular localization studies showed that the different AtG3BPs are also localized in SG upon heat stress. In addition, AtG3BP-2 localizes into SGs, if the plant cell is treated with KCN^9^. Kedersha and colleagues^37^ concluded that G3BP binds 40S ribosomes through its RGG region and is essential for stress granule condensation, but distinct from polysome disassembly, and the condensation process is regulated by competitive Caprin1/USP10 binding to G3BP. The *A. thaliana* UBP-24 protein is the plant homolog of human USP10^9^. Consequently, all putative members of the AtG3BP family studied interact *in planta* with AtUBP-24. In addition, homo- and heterodimerization, as described for the human G3BPs^2^, was also confirmed in this study. The analyzed AtG3BP family members may, therefore, fulfill a similar role in SG assembly and disassembly as their well investigated mammalian counterpart and thus may also play a pivotal role in viral infection^15,16^, as previously suggested by interaction studies of AtG3BP-2 and the nuclear shuttle proteins of ssDNA plant viruses^9^. Therefore, it was necessary to investigate if the other identified members of the AtG3BP family were also affected by a virus infection. First, tissue tropism and expression level of the AtG3BPs was determined, and then the question was addressed if the expression level alters upon virus infection. Not surprisingly, six of seven AtG3BP members were significantly upregulated in infected leaf tissue, but most interestingly only AtG3BP-4 was significantly induced in early infection, as shown by transcriptomic data obtain from dissected tissue and confirmed by qRT-PCR analysis. The complete analysis of the TuMV differentially induced expressed genes in *A. thaliana* tissue at 2 dpi will be described elsewhere and further analyses on the role of AtG3BP-4 in early and systemic virus infection, in general, must and will be performed in the future. It is reasonable to assume that G3BP plays a role at an early stage of infection; for example, members of the *Picornaviridae* family also modulate SGs accumulation during replication. Poliovirus regulates SGs in a time-dependent manner; at early times the 2A proteinase induces assembly of SGs that are later disassembled by the 3C proteinase through G3BP-1 cleavage^38^. Similar temporal control of SG assembly is exhibited by Coxsackievirus B3 assembly as early as 3 h post-infection.

Given that SGs are generally associated with regulation of gene expression, viruses have evolved different mechanisms to counteract their assembly or to use them in their favor to replicate within the host environment successfully. In this study, data is presented about a SG key factor, namely G3BP. As their mammalian equivalents, the AtG3BPs behave similarly and their expression in early response to a virus infection was investigated and confirmed. Particular focus must, therefore, be on AtG3BP-4, which we show is highly upregulated upon TuMV infection. Analysis of KO and OEX plant lines in the future will shed more light on the G3BP family's role in virus infection in particular and other stresses in general.

## Methods

### Plant material and growth conditions

*Arabidopsis thaliana* ecotype Columbia (Col-0; NASC ID N1092) and *AtSUC2*promoter::GFP plants (as described in^39^) were used throughout the study. *A. thaliana* plants were grown on soil in a growth chamber at 24 °C under short-day conditions (8 h light/16 h dark). *Nicotiana benthamiana* plants were grown in an insect-free greenhouse under long-day conditions (16 h supplementary light) at 22 °C.

### Expression plasmid construction

The coding regions of AtG3BP-1 (AT5G60980), AtG3BP-2 (AT5G43960), AtG3BP-3 (AT3G25150), AtG3BP-4 (AT1G69250), AtG3BP-5 (AT1G13730), AtG3BP-6 (AT2G03640), AtG3BP-7 (AT5G48650), AtUBP-24 (AT4G30890) and PNYDV Clink (Accession JN133280) were amplified by PCR and inserted into the vector pENTR™/D-TOPO™ (Invitrogen). For C-terminal fusion constructs the translational stop codon was removed by PCR. The inserts were then transferred into the expression vectors pGWB441^40^, pB4nYGW, pB4cYGW, pB4GWnY, pB4GWcy^41^, pRB-C-Venus^N173^ and pRB-C-Venus^C155^^42^ using L/R-Clonase™ II enzyme mix (Invitrogen) according to the manufacturer’s protocol. The resulting plasmids are expressing the corresponding genes in fusion with the reporter gene under the control of the CaMV 35S promoter. All destination vectors and the used primers are listed in Supplementary Table S1. All plasmids were confirmed by Sanger sequencing (Eurofins, Ebersberg, Germany).

### Transient expression of fusion proteins in *N. benthamiana*

All expression plasmids were transformed into *Agrobacterium tumefaciens* strain LBA4404 or GV3101 by electroporation. *Agrobacterium* cultures were grown in selective media and infiltrated at a final OD_600_ = 0.1. For bimolecular fluorescence complementation (BiFC) experiments cultures were mixed and then co-infiltrated at a 1:1 ratio into the abaxial surface of 3–5 week-old *N. benthamiana* leaves.

### Heat treatment of plant samples and microscopy

Heat stress was applied by incubating the samples for 45 min at 37 °C in a humidified box. Fluorescence was visualized in epidermal cell layers of the leaves after 2–3 days of infiltration using confocal microscopy. A Leica TCS SP8 confocal laser-scanning microscope was utilized for visualization of EYFP and BiFC signals. Enhanced yellow fluorescent protein (EYFP) and VENUS were excited by using a 488 and 514 nm laser. A Leica HC PL APO CS2 63×/1.20 (NA = 0.75) water objective was employed. BiFC images were acquired with a resolution of 1024 × 1024 pixels. Images for the analyses of the cellular localization were acquired with a resolution of 2632 × 2632 pixels for deconvolution with the SVI Huygens Essential software (Scientific Volume Imaging B.V., Hilversum, Netherlands) embedded in the Leica LAS X program (Leica Microsystems, Wetzlar, Germany) using the standard strategy. Images were processed and compiled using Photoshop CS4 (Adobe, San Jose, CA).

### Western blot analysis

Proteins were extracted from 100 mg infiltrated leaf material with 250 µl SDS-sample buffer (4% SDS, 20% glycerol, 5% β-Mercaptoethanol, 100 mM Tris–HCl pH 6.8, 0.005% bromophenol blue) after grinding in liquid nitrogen. The extracts were then centrifuged at 13.000 rpm for 10 min and the supernatants were collected. Samples were resolved in a 4–20% Mini-PROTEAN® TGX™ Precast Protein Gel (Bio-Rad, Hercules, CA) and transferred to an Amersham™ Hybond™ P 0.45 PVDF membrane (GE Healthcare, Germany). PVDF membranes were blocked using 5% skim milk powder in TBS-T (0.05% Tween 20) and incubated with primary antibody (Mouse anti-GFP; Invitrogen, GF28R, 1:2000) for 16 h at 4 °C followed by incubation with HRP-linked secondary antibody (Goat anti-Mouse HRP-conjugated; Invitrogen, 32,430, 1:500) (1 h at room temperature) in TBS-T. Chemiluminescence was detected using SuperSignal West Pico substrate (Thermo Fisher Scientific, Germany) and a ChemiDoc XRS + Imaging system (Bio-Rad, Hercules, CA).

### Sap inoculation of experimental plants

The mechanical inoculation of *A. thaliana* Col-0 plants was performed by grinding TuMV-RFP^29^ infected leaf material in homogenization buffer (0.05 M K/Na-phosphate, 1 mM EDTA, 5 mM Na-DIECA, 5 mM thioglycolic acid; pH 7.0) and rubbing onto the Celite-covered abaxial surface of healthy 4-week-old *A. thaliana* plants. As a control, mock inoculations with buffer only were performed. Since we were also interested in sampling early infection sites such as two dpi, we used eight-weeks-old *A. thaliana* plants with fully developed leaves to avoid tissue damage during rub inoculation in small seedlings.

### Laser dissection microscopy

Samples of *AtSUC2*promoter::GFP plant petioles or TuMV-RFP^29^ infection sites, respectively, were fixed in 10% formalin under vacuum for 15 min and left shaking in fixative at 4 °C overnight. Fixed samples were then washed twice in PBS for 15 min at 4 °C before being vacuum infiltrated with ice-cold 10% sucrose followed by 20% sucrose. The cryo-protected leaves are then embedded in OCT compound within molds and immediately frozen in isopentane cooled with liquid nitrogen. Cryosections were carried out in a Cryostar NX50 (Thermo Fisher Scientific, Waltham, MA). 10 µm thick sections were mounted onto membrane slides (Molecular Machines and Industries, Eching, Germany) and washed with distilled water and dehydrated in pre-chilled ethanol grades (70%, 95%, and 100%) and air-dried. Fluorescent cells were then collected with a CellCut laser microdissection microscope (MMI, Eching, Germany). The slides were observed using a CFI S Plan Fluor ELWD 20x (NA = 0.45) objective with UV light excitation. Extraction was performed employing the following laser settings: Cut velocity 15 µm/s; Laser focus 158 µm; Laser power 85%. For phloem analysis, approximately 1 mm^2^ of GFP-labelled phloem cells were pooled in a 0.2 ml Isolation cap microcentrifuge tube (MMI, Eching, Germany).

RNA was extracted from plant material with the GenElute FFPE RNA Purification Kit (Sigma, Germany) according to the manufacturer’s protocol with modifications. The Deparaffinization step was omitted since samples were embedded in OCT. 30 μl Digestion Buffer A and 1 μl of the reconstituted Proteinase K were added to the cap and incubated for 30 min at 55 °C with vortexing each 10 min. The tubes were inverted. After a spin down, samples were transferred to a new 1.5 ml microcentrifuge tube, and another 270 μl of Digestion Buffer A and 9 μl of Proteinase K were added. The tube was incubated for 15 min at 55 °C with vortexing each 5 min.

We used laser microdissection to collect only infection sites with minimum non-infected surrounding tissue (CellCut laser microdissection microscope, MMI, Eching, Germany). Small pieces (~ 0.25 mm^2^) of infected and mock leaves were mounted onto membrane slides (Molecular Machines and Industries, Eching, Germany) and covered with 70% ethanol for 10 min for a quick fixation and then with 100% ethanol until the ethanol evaporated from the slide. This step will reduce the water content of the leaves and facilitate the sectioning. RFP-labeled infection sites were precisely dissected, and the similar dissected area was obtained from mock-inoculated plants using the fluorescence module of the CellCut laser microdissection microscope. Three infection sites per plant were collected and pooled in the same 0.2 ml cap, and five replicates were obtained.

### RNA isolation and qRT-PCR analysis

Total RNA from *A. thaliana* rosette leaves, stem, siliques, flowers, and roots was extracted using the RNeasy Plant Mini Kit (Qiagen, Hilden, Germany) according to the manufacturer’s protocol. RNA extracts were treated with RNase-free DNase I and quantified with a NanoDrop2000 spectrophotometer. For cDNA synthesis, 1.5 µg of total RNA were transcribed with 300 U M-MLV Reverse Transcriptase using oligo (dT)_12–18_ primer (0.5 µM). For each 20 µl qRT-PCR reaction 1 µL of cDNA, 10  µl of KAPA SYBR® FAST qPCR Master Mix (2X), and primers (0.2 µM) specific for AtActin-2 (AT3G18780), AtG3BP-1 (AT5G60980), AtG3BP-2 (AT5G43960), AtG3BP-3 (AT3G25150), AtG3BP-4 (AT1G69250), AtG3BP-5 (AT1G13730), AtG3BP-6 (AT2G03640), and AtG3BP-7 (AT5G48650) were used. Reactions were run on a qTOWER^3^ G (Analytik Jena, Jena, Germany) and a Mastercycler® realplex (Eppendorf, Hamburg, Germany). Between 6 and 12 biological replicates, with 3 technical replicates each, were used in every experiment. Transcript levels and ratios were then calculated using the 2^−∆Ct^ or the 2^−∆∆Ct^ method, respectively, with *AtActin-2* as the endogenous control. Statistical significance was measured by two-tailed t-test. The primers utilized for the detection of specific transcripts are listed in Supplementary Table S2.

### Library preparation, characterization and sequencing

Individual libraries from mock and TuMV-RFP-infected plants were prepared according to the Smart-3SEQ protocol previously described in^43^. The quality of libraries was checked with Agilent 2200 using the high sensitive DNA chip from Agilent. Libraries were sequenced in the NextSeq 500 using the NextSeq 500/550 high output Kit v2.5, 75 cycles from Illumina (Illumina Inc., San Diego, US).

### Data preprocessing

After sequencing, reads were demultiplexed and converted to FASTQ with bcl2fastq (Illumina Inc., San Diego, US) with the adapters trimmed. UMI sequence, G-overhang, and A-tails from FASTQ data were removed with the script umi_homopolymer.py as described by Ref.^43^. Reads shorter than 18 nt were removed with the tool Filter FASTQ^44^.

### Read alignment, counting and analysis of gene expression

Final reads were mapped to the genome of *A. thaliana* Col-0 using Bowtie2 tool using the default setting^45^. Resulting BAM files from Bowties2 were used to count the reads with the tool featureCounts^46^. Gene differential expression between mock and TuMV-RFP-infected plants was calculated using output files from featureCounts with the DESeq2 tool^47^.

### Phylogenetic analysis

AtG3BP protein and coding sequences were retrieved from the TAIR10 genome annotation data set (http://www.arabidopsis.org/). Multiple sequence alignments were carried out in MEGA X software utilizing the CLUSTAL W algorithm^48^. A BLOSUM62 matrix was used with a gap opening penalty of 10 and a gap extension penalty of 0.2. All phylogenetic trees were constructed with the Neighbor Joining (NJ) method^49^ with 1000 bootstrap replicas^50^ using the Poisson correction method^51^ and are in the units of the number of amino acid substitutions per site. Protein domains were identified using ExPASy PROSITE (https://prosite.expasy.org/)^52^. Geneious Prime® 2020.1.2 (https://www.geneious.com) was used to visualize alignments and protein domains.

## Supplementary Information

Supplementary Information 1.

Supplementary Information 2.
