# Supplementary material for: Analyzing the G3BP-like gene family of Arabidopsis thaliana in early turnip mosaic virus infection
Source: Sci Rep. 2021 Jan 26;11:2187. doi: 10.1038/s41598-021-81276-7 (PMC7838295; doi:10.1038/s41598-021-81276-7)
Supplement: Supplementary file 1 — Supplementary Information 1. [file 41598_2021_81276_MOESM1_ESM.docx]

Analyzing the G3BP-like gene family of Arabidopsis thaliana in early turnip mosaic virus infection Hendrik Reuper, Khalid Amari and Björn Krenz


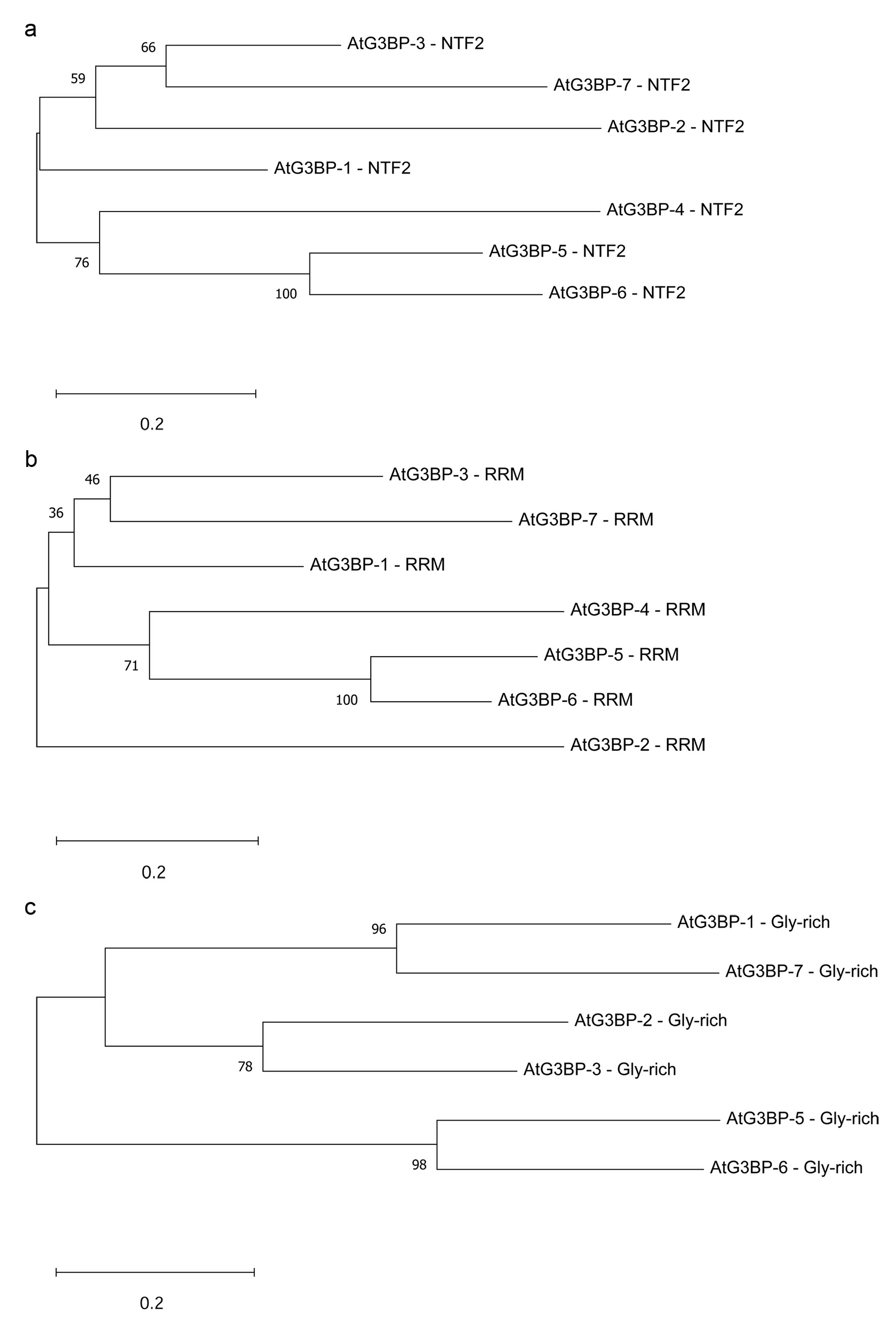


**Supplementary Figure S1** Phylogenetic analysis of the different domains of the AtG3BP family.

The optimal tree with the sum of branch length of (a) 2.60190885 for the NTF2-like domain, (b) 2.49910748 for the RRM domain and (c) 2.64850966 for the Gly-rich region is shown. Evolutionary analyses were carried out as described in the methods section.


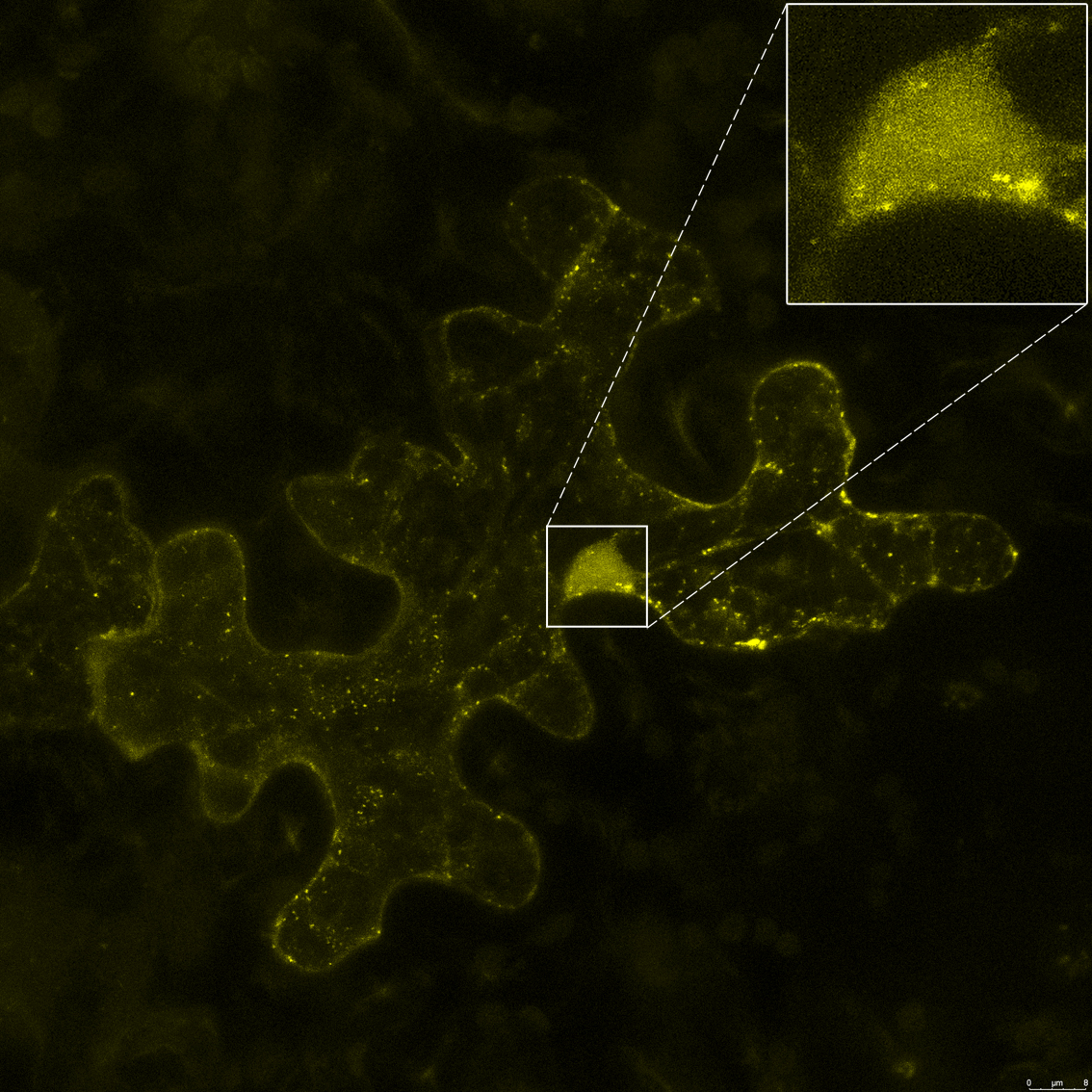


**Supplementary Figure S2** Sub-cellular localization of AtG3BP-6::EYFP transient expressed in *N. benthamiana* leaves after heat shock. The enlarged area highlights the nuclear localization of AtG3BP-6. The picture is a maximum projection of a z-stack obtained by confocal laser scanning microscopy and was taken 2 dpai. The picture corresponds to a size of 145 x 145 µm.


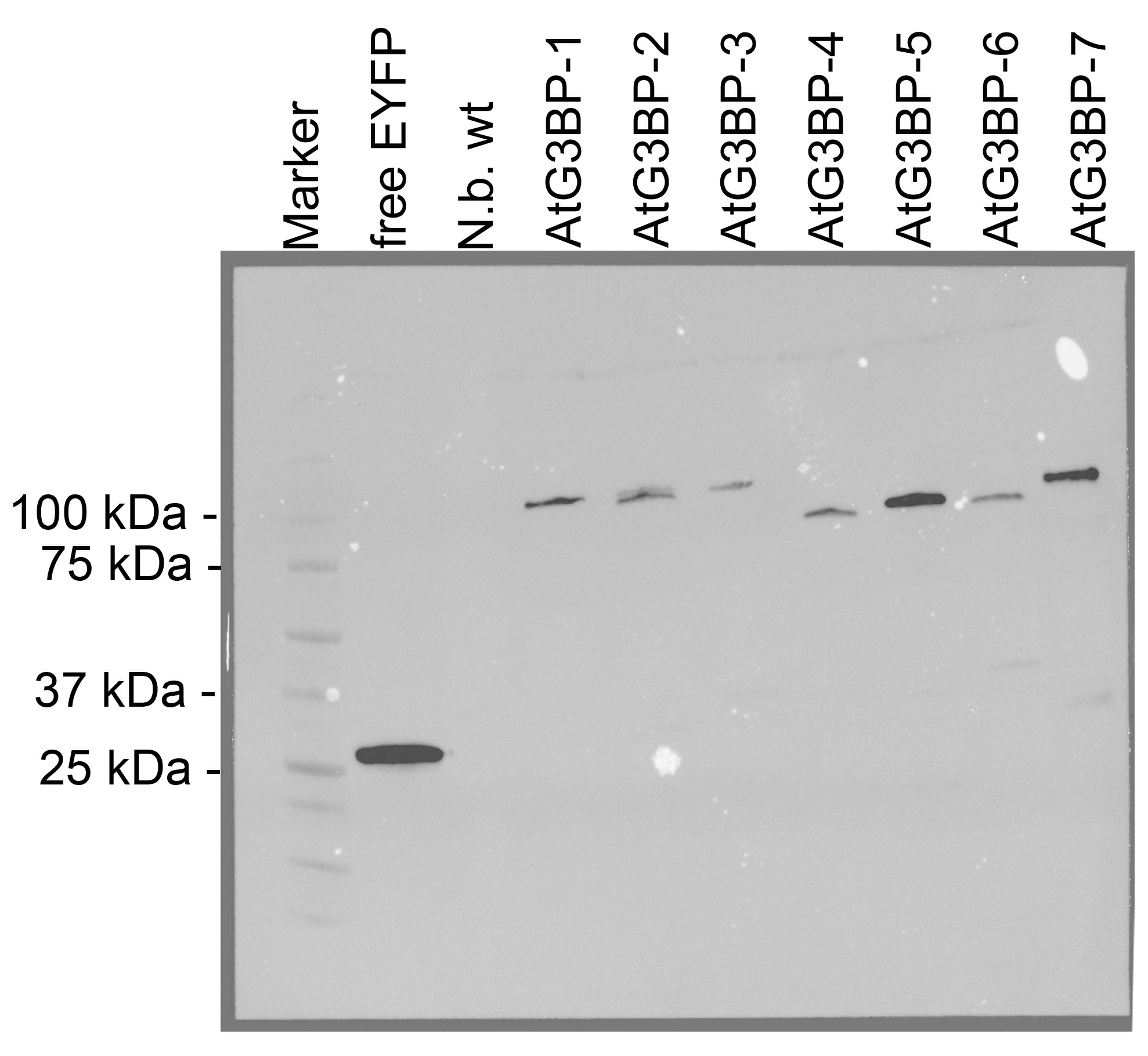


**Supplementary Figure S3** Expression control of AtG3BP::EYFP fusion proteins. Protein extracts from infiltrated *N. benthamiana* leaves were separated via PAGE and immunoblotted against EYFP with a mouse anti-GFP antibody as described in the methods section. *N. benthamiana* leaves transiently expressing free EYFP were used as positive control, un-infiltrated leaves as negative control.


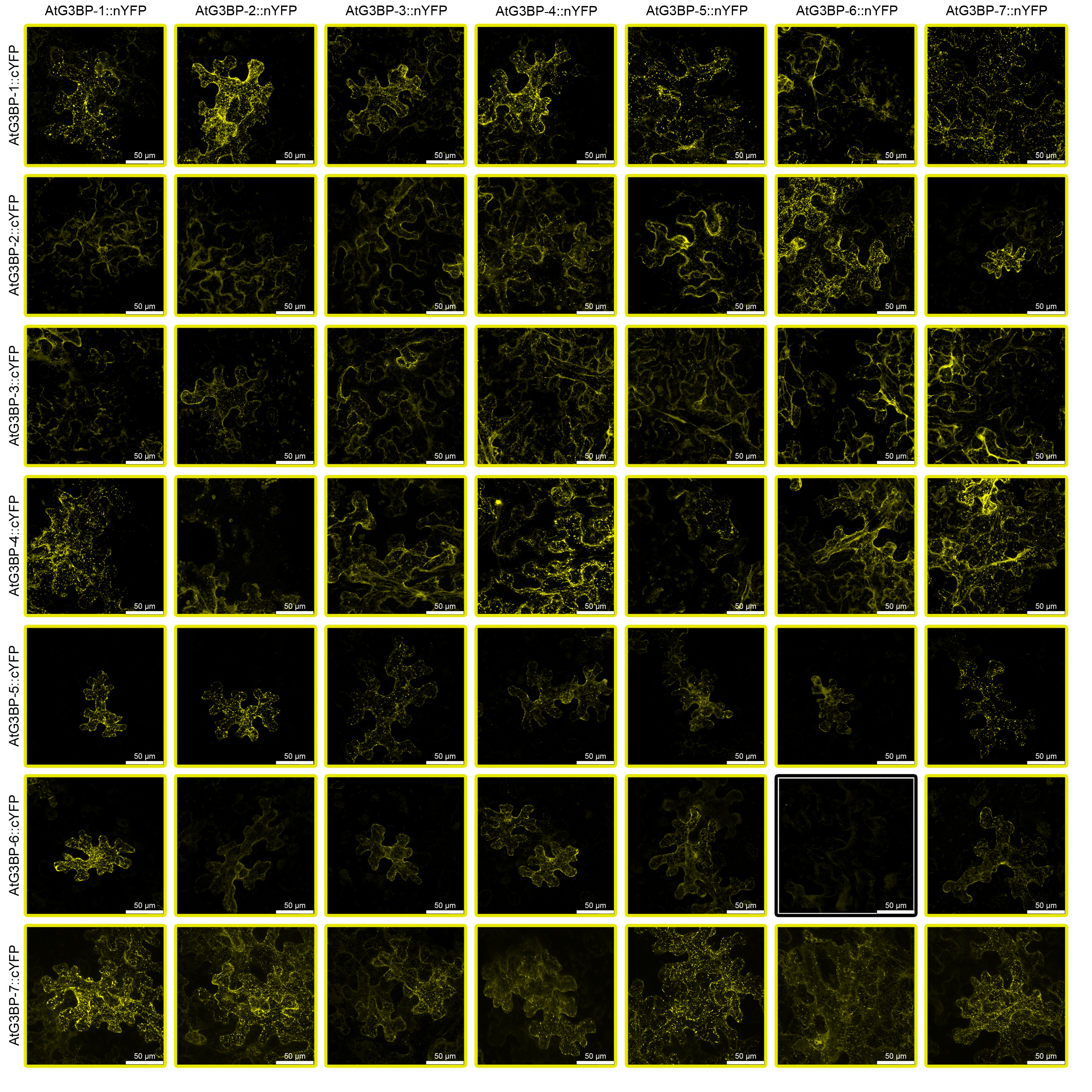


**Supplementary Figure S4** BiFC analysis of AtG3BPs C-terminally fused to splitYFP under ambient conditions. Yellow frames indicate interaction, black frames no interaction. Possible yellow spots in the black framed panels are due to increased gain of the detector to detect even very low signals. The images displaying the self-interaction of the respective AtG3BP::splitYFP constructs are the same as in Figure 3A.


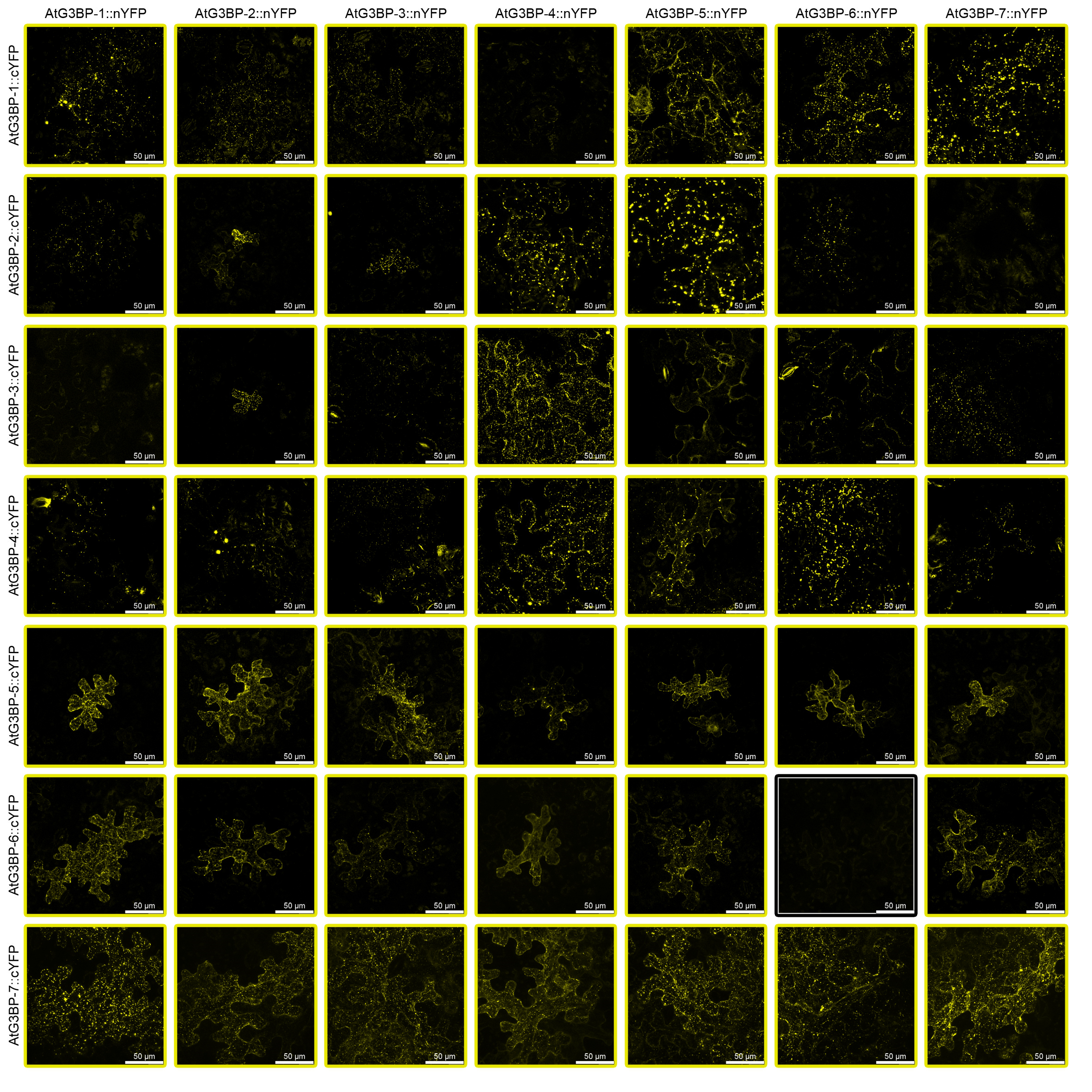


**Supplementary Figure S5** BiFC analysis of AtG3BPs C-terminally fused to splitYFP after heat shock. Yellow frames indicate interaction, black frames no interaction. Possible yellow spots in the black framed panels are due to increased gain of the detector to detect even very low signals. The images displaying the self-interaction of the respective AtG3BP::splitYFP constructs are the same as in Figure 3A.


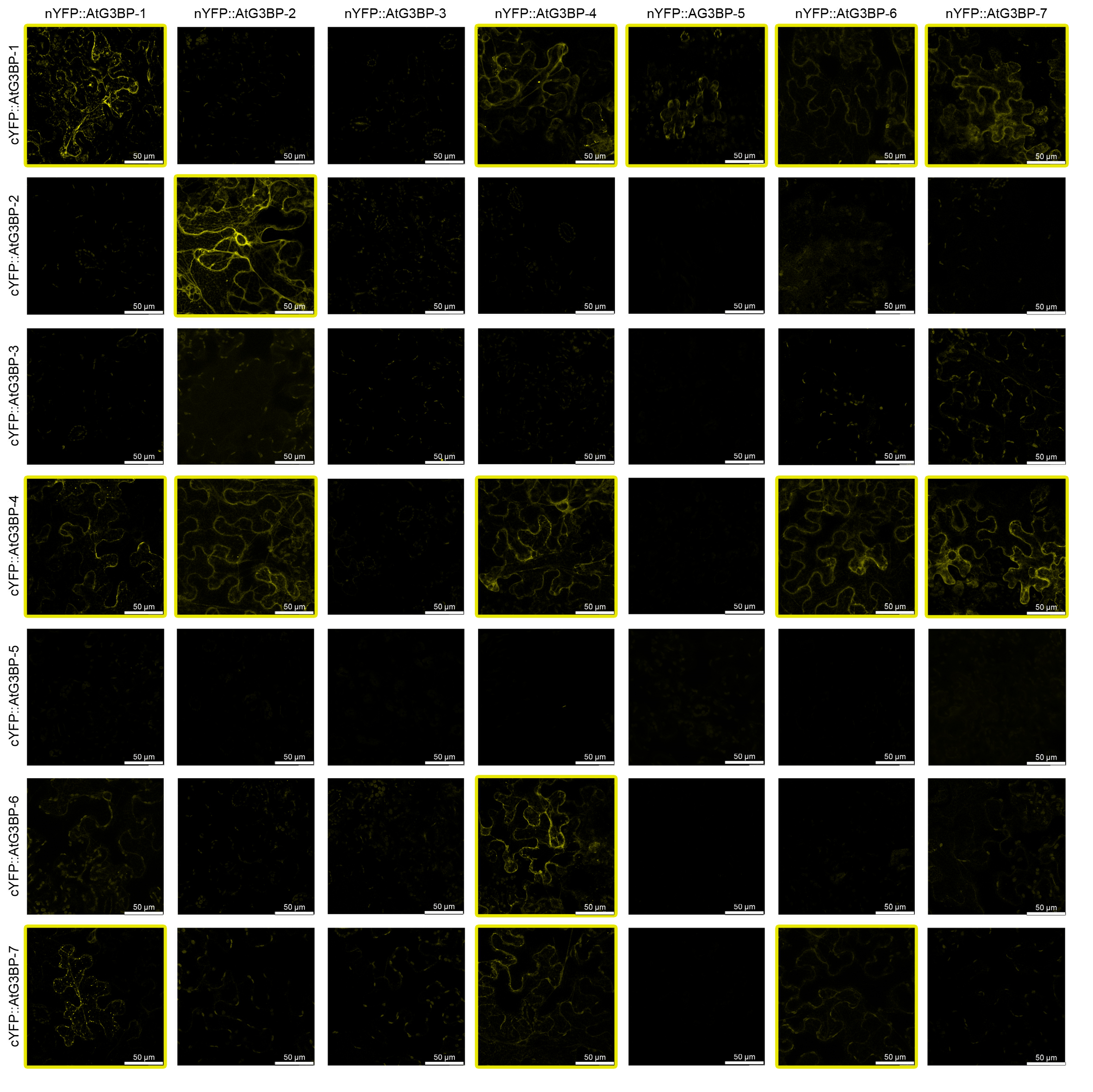


**Supplementary Figure S6** BiFC analysis of AtG3BPs N-terminally fused to splitYFP under ambient conditions. Yellow frames indicate interaction. Possible yellow spots in the none-framed panels are due to increased gain of the detector to detect even very low signals. The images displaying the self-interaction of the respective splitYFP::AtG3BPs are the same as in Figure 3B.


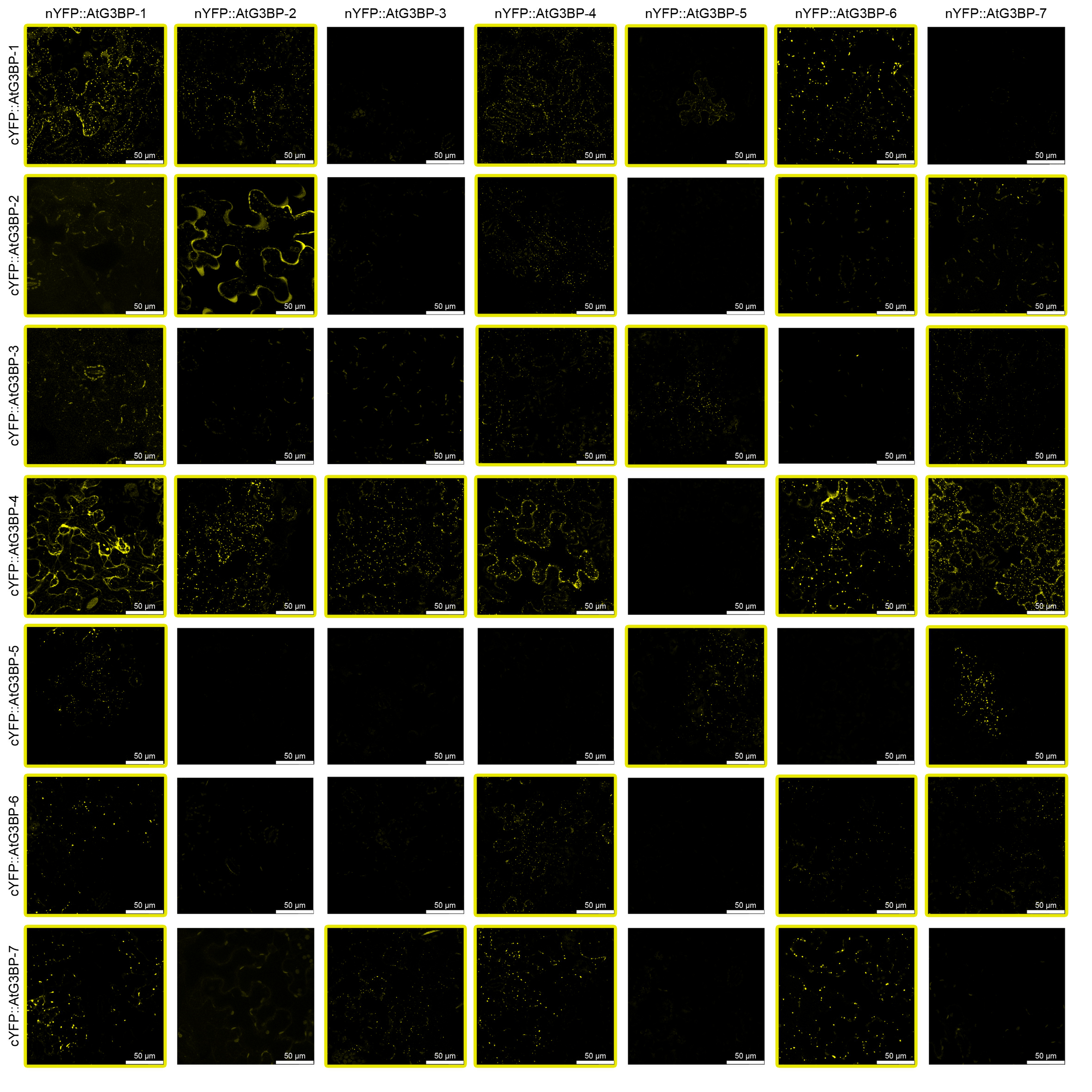


**Supplementary Figure S7** BiFC analysis of AtG3BPs N-terminally fused to splitYFP after heat shock. Yellow frames indicate interaction. Possible yellow spots in the none-framed panels are due to increased gain of the detector to detect even very low signals. The images displaying the self-interaction of the respective splitYFP::AtG3BPs are the same as in Figure 3B.


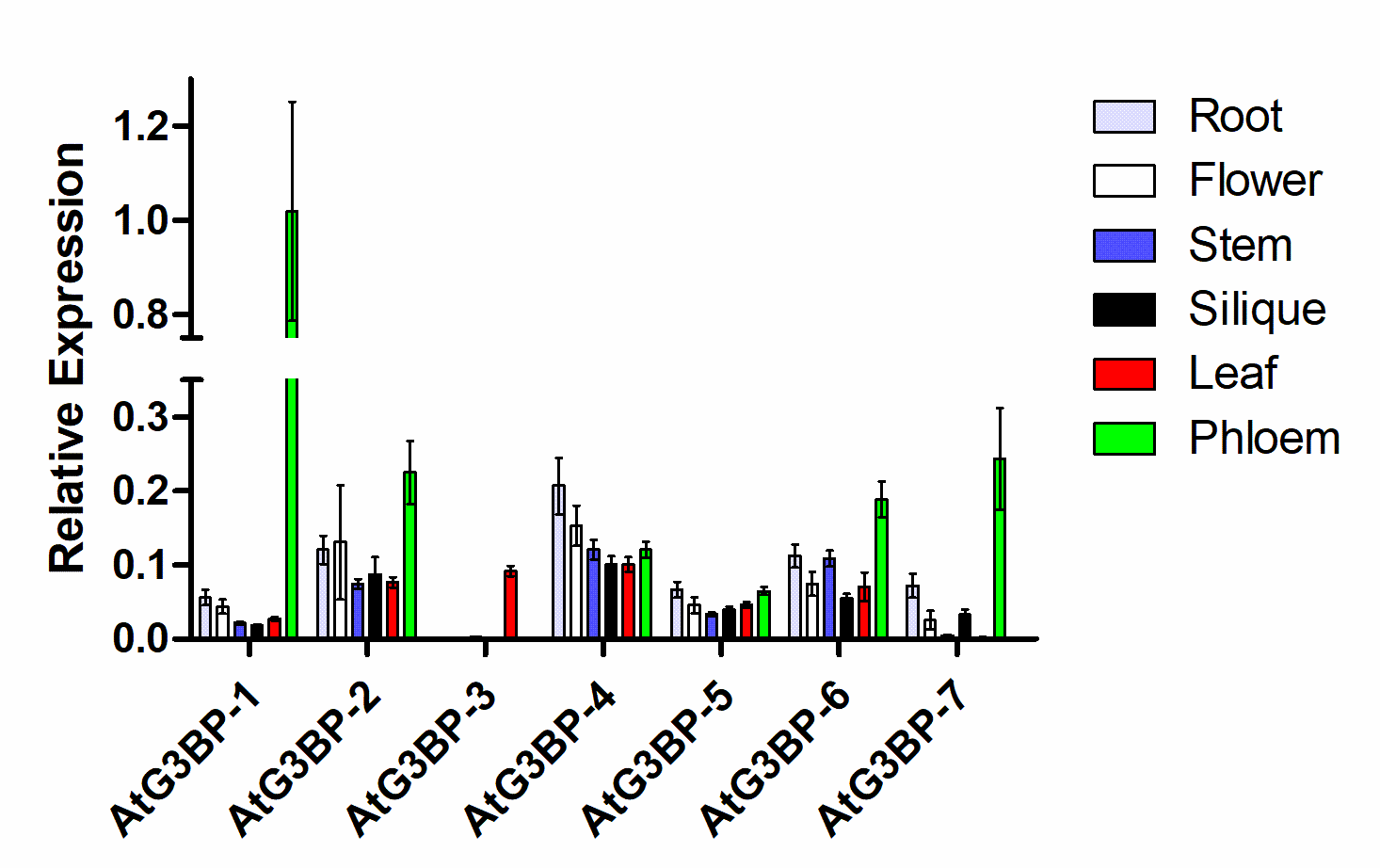


**Supplementary Figure S8** Expression level analysis. (a) qRT-PCR was used to measure the relative gene expression of all *AtG3BP*s in roots, stem, flower, silique, leaves and laser dissected phloem tissue. Transcript levels were calculated using the 2^-∆Ct^ method with *AtActin-2* as the endogenous control. The error bar represents the standard error. The different extraction method for phloem tissue and therefore limited comparability must be noted here.


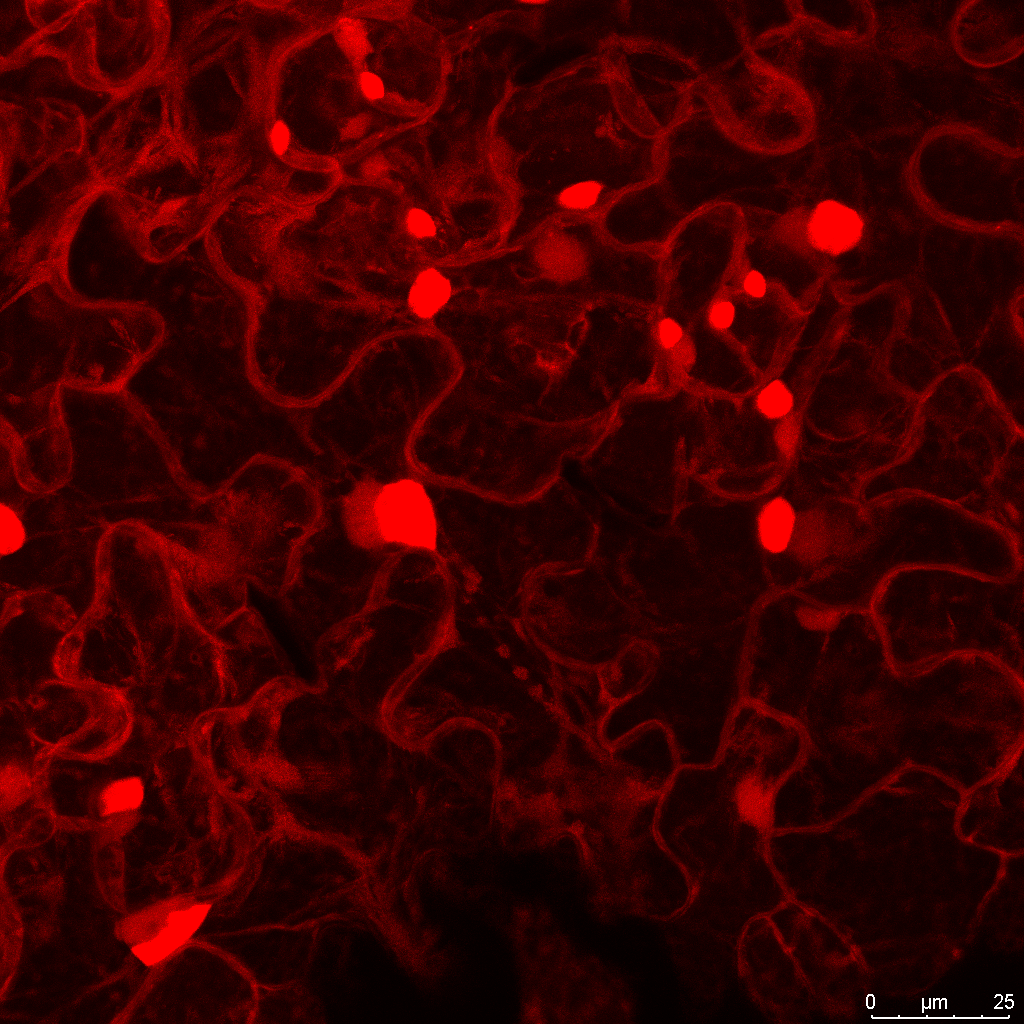


**Supplementary Figure S9** Systemic TuMV-RFP infection in *Arabidopsis thaliana.* The picture was obtained 14 dpi by confocal laser scanning microscopy and is a projection of a z-stack.

**Supplementary Table S1** List of used Gateway destination vectors

| Gateway destination vectors | Reporter | Antibiotic resistance | Reference |
| --- | --- | --- | --- |
| pRB35S-C-Venus^N173^-GW | C-terminal Venus^N173^ | Spectinomycin and Streptomycin | Nietzsche et al., 2014 |
| pRB35S-C-Venus^C155^-GW | C-terminal Venus^C155^ | Spectinomycin and Streptomycin | Nietzsche et al., 2014 |
| pB4nYGW | N-terminal nYFP | Spectinomycin | Kamigaki et al., 2016 |
| pB4cYGW | N-terminal cYFP | Spectinomycin | Kamigaki et al., 2016 |
| pB4GWnY | C-terminal nYFP | Spectinomycin | Kamigaki et al., 2016 |
| pB4GWcY | C-terminal cYFP | Spectinomycin | Kamigaki et al., 2016 |
| pGWB 441 | C-terminal EYFP | Spectinomycin | Nakagawa et al., 2007 |

**Supplementary Table S2** List of used Oligonucleotides

| **Primer** | **Gene** | **Sequence 5'-3'** |
| --- | --- | --- |
| G3BP-1-fw | AtG3BP-1 (AT5G60980) | CACCATGGCACAGCAGGAA |
| G3BP-1-rev | AtG3BP-1 (AT5G60980) | TCAAGATGAACCACCACCTCGAG |
| G3BP-1-rev-nS | AtG3BP-1 (AT5G60980) | AGATGAACCACCACCTCGAG |
| G3BP-2-fw | AtG3BP-2 (AT5G43960) | CACCATGGCGACTCCTTATCCT |
| G3BP-2-rev | AtG3BP-2 (AT5G43960) | TTAGCGACCACCACCGCG |
| G3BP-2-rev-nS | AtG3BP-2 (AT5G43960) | GCGACCACCACCGCGGTA |
| G3BP-3-fw | AtG3BP-3 (AT3G25150) | CACCATGGCGATGTTAGGTGCACAGCAAGTT |
| G3BP-3-rev | AtG3BP-3 (AT3G25150) | TTACGCAGCAACAGACACACG |
| G3BP-3-rev-nS | AtG3BP-3 (AT3G25150) | CGCAGCAACAGACACACGG |
| G3BP-4-fw | AtG3BP-4 (AT1G69250) | CACCATGGCTACCGAGGGAG |
| G3BP-4-rev | AtG3BP-4 (AT1G69250) | TCAATGTGCGGCTTCACTTTTT |
| G3BP-4-rev-nS | AtG3BP-4 (AT1G69250) | ATGTGCGGCTTCACTTTTTTTCC |
| G3BP-5-fw | AtG3BP-5 (AT1G13730) | CACCATGGCACTTGAATCAAATGCT |
| G3BP-5-rev | AtG3BP-5 (AT1G13730) | TTAACGGCTAGCTTCTACCGTACC |
| G3BP-5-rev-nS | AtG3BP-5 (AT1G13730) | ACGGCTAGCTTCTACCGTACC |
| G3BP-6-fw | AtG3BP-6 (AT2G03640) | CACCATGACACCTGAATCAAACGC |
| G3BP-6-rev | AtG3BP-6 (AT2G03640) | CTAGTTTTTGGCCTCAGTATTGCC |
| G3BP-6-rev-nS | AtG3BP-6 (AT2G03640) | GTTTTTGGCCTCAGTATTGCCATG |
| G3BP-7-fw | AtG3BP-7 (AT5G48650) | CACCATGGATTCTACTGCTGCAAC |
| G3BP-7-rev | AtG3BP-7 (AT5G48650) | CTAGTACGAGTTGATGCTGGCGA |
| G3BP-7-rev-nS | AtG3BP-7 (AT5G48650) | GAGTTGATGCTGGCGACGTA |
| UBP24-fw | AtUBP-24 (AT4G30890) | CACCATGAGTGAAAAGAAGGTATTTGTGTTTG |
| UBP24-rev-nS | AtUBP-24 (AT4G30890) | CACTTGCTTGTAGAAGAGGACATAGGC |
| qAt-Actin2-fw | AtActin-2 (AT3G18780) | CACCACAACAGCAGAGCGGGA |
| qAt-Actin2-rev | AtActin-2 (AT3G18780) | TCCCACAAACGAGGGCTGGA |
| qAtG3BP1-fw | AtG3BP-1 (AT5G60980) | TTCACCAATCTCCCGGTTTAG |
| qAtG3BP1-rev | AtG3BP-1 (AT5G60980) | CGTTGATCGCTTGCATAGTTG |
| qAtG3BP2-fw | AtG3BP-2 (AT5G43960) | TCTGCAAGAGGACCAACATC |
| qAtG3BP2-rev | AtG3BP-2 (AT5G43960) | CCACATCAACTGCAACTTCTTC |
| qAtG3BP3-fw | AtG3BP-3 (AT3G25150) | CAGTGTCAGGAGGACGTTTAG |
| qAtG3BP3-rev | AtG3BP-3 (AT3G25150) | TGTACGACAGTGCCTTCATC |
| qAtG3BP4-fw | AtG3BP-4 (AT1G69250) | AGACCGTAAGCTTCGTGTAAAG |
| qAtG3BP4-rev | AtG3BP-4 (AT1G69250) | TGCTATCTGCAGAGCCATTC |
| qAtG3BP5-fw | AtG3BP-5 (AT1G13730) | GTTACAGGCTCAAGGGAAACT |
| qAtG3BP5-rev | AtG3BP-5 (AT1G13730) | CCGATTCTGATGGCTGATTCT |
| qAtG3BP6-fw | AtG3BP-6 (AT2G03640) | CTGTATTGGGTTTGTGGCATTC |
| qAtG3BP6-rev | AtG3BP-6 (AT2G03640) | GCTCTTCGGTTTCCGATTCT |
| qAtG3BP7-fw | AtG3BP-7 (AT5G48650) | GTGAAGATTGGTGGACTGAGAG |
| qAtG3BP7-rev | AtG3BP-7 (AT5G48650) | CCCACTCCCACATTCCTATTT |
